# What’s Needed to Develop Strategic Purchasing in Healthcare? Policy Lessons from a Realist Review

**DOI:** 10.15171/ijhpm.2018.93

**Published:** 2018-10-02

**Authors:** Joe Sanderson, Chris Lonsdale, Russell Mannion

**Affiliations:** ^1^Birmingham Business School, University of Birmingham, Birmingham, UK.; ^2^Health Services Management Centre, University of Birmingham, Birmingham, UK.

**Keywords:** Strategic Purchasing, Healthcare, Complementary Theories

## Abstract

**Background:** In the context of serious concerns over the affordability of healthcare, various authors and international policy bodies advise that strategic purchasing is a key means of improving health system performance. Such advice is typically informed by theories from the economics of organization (EOO). This paper proposes that these theories are insufficient for a full understanding of strategic purchasing in healthcare, because they focus on safeguarding against poor performance and ignore the coordination and adaptation needed to improve performance. We suggest that insights from other, complementary theories are needed.

**Methods:** A realist review method was adopted involving 3 steps: first, drawing upon complementary theories from the EOO and inter-organizational relationships (IOR) perspectives, a theoretical interpretation framework was developed to guide the review; second, a purposive search of scholarly databases to find relevant literature addressing healthcare purchasing; and third, qualitative analysis of the selected texts and thematic synthesis of the results focusing on lessons relevant to 3 key policy objectives taken from the international health policy literature. Texts were included if they provided relevant empirical data and met specified standards of rigour and robustness.

**Results:** A total of 58 texts were included in the final analysis. Lessons for patient empowerment included: the need for clearly defined rights for patients and responsibilities for purchasers, and for these to be enacted through regular patientpurchaser interaction. Lessons for government stewardship included: the need for health strategy to contain specific targets to incentivise purchasers to align with national policy objectives, and for national government actors to build close, trusting relationships with purchasers to facilitate access to local knowledge about needs and priorities. Lessons for provider performance included: provider decision autonomy may drive innovation and efficient resource use, but may also create scope for opportunism, and interdependence likely to be the best power structure to incentivise collaboration needed to drive performance improvement.

**Conclusion:** Using complementary theories suggests a range of general policy lessons for strategic purchasing in healthcare, but further empirical work is needed to explore how far these lessons are a practically useful guide to policy in a variety of healthcare systems, country settings and purchasing process phases.

## Background

In the context of serious concerns over the current and future affordability of healthcare,^1-4^ various authors and international policy bodies have identified strategic purchasing as a potentially significant driver of improved healthcare system performance assessed in terms of responsiveness to patient needs, equity of access and efficient resource utilisation.^5-10^

**Figure 1:**
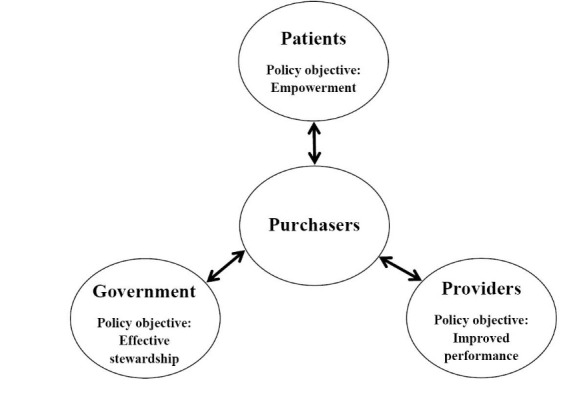
Key Actors, Relationships and Policy Objectives Needed to Develop Strategic Purchasing in Healthcare.

Strategic purchasing has been defined by the World Health Organization (WHO) as a process that goes beyond a passive and relatively unsystematic allocation of funds to healthcare providers to encompass ‘a continuous search for the best interventions to purchase, the best providers to purchase from, and the best payment mechanisms and contracting arrangements to pay for such interventions’ (p. 105).^7^ Strategic purchasing is thus much more than the simple financing of healthcare services. It involves an evaluation of population health needs, the planning and design of healthcare services, the qualification and selection of appropriate providers, and the incentivization and management of providers to ensure good performance. Consistent with this definition it is suggested by several leading contributors to the international health policy literature that the development of strategic healthcare purchasing should be focused on 3 interrelated relationships between 4 groups of actors: (1) patients and purchasers; (2) government and purchasers; and (3) purchasers and providers.^6,9^ These authors suggest, in turn, that 3 key policy objectives associated with these actors and relationships should be pursued: patient empowerment; effective government stewardship; and improved provider performance (see Figure 1).

Of course, although strategic purchasing in different country settings needs to address these 3 key objectives, it will not create one common set of organizational structures or one type of relationship behaviour. Indeed, a diversity of structures, behaviours and funding mechanisms has developed,^3,5^ shaped by the particularities of each country’s institutions and political system.^6^ However, this commonality of objectives still means that policy-makers should be encouraged to learn broad lessons from other countries to understand how healthcare purchasing might become more strategic in their own setting.

Crucially, in order for such learning to go beyond uncritical notions of lesson drawing and policy transfer, it must be theoretically informed.^11,12^ This is so that the likelihood of a policy reform being translated from one setting to another, and the form which that translation might take, can be systematically understood.^13^ Theories are important for understanding how decision-makers might behave in different settings and how particular policy reforms might be interpreted and implemented.^14^ Moreover, theories are intensely practical and should be used by practitioners as guidance in deciding what to do and how to do it.^15^

To develop this point, it has been observed that mainstream Neoclassical economic theory has played a particularly influential role in shaping healthcare purchasing reforms over recent decades.^16^ This use of theory has not been without criticism, however, given that it has led policy-makers to adopt rather superficial notions of demand, supply and market competition.^17,18^ As part of attempts to look beyond such ideas, the economics of organization (EOO) literature encompassing agency theory (AT) and transaction cost economics (TCE) has been suggested as a more sophisticated and relevant perspective.^5,6,19^

There is indeed evidence that this EOO literature can offer policy lessons about the contractual and extra-contractual governance structures through which relationships between patients, government, purchasers and providers might be safeguarded against the risks of bad behaviour and poor performance.^5,19^ Despite these insights, though, we argue that this literature provides an incomplete basis for a model of strategic purchasing in healthcare. This is because the strategic purchasing research domain is also concerned with relationship coordination and adaptation to achieve effective performance not just with safeguarding against the risk of poor performance.^20^ A more complete understanding of how strategic purchasing might be achieved thus requires an engagement with other theories.

We argue that the inter-organizational relationships (IOR) literature, which addresses notions like relational capabilities, norms and trust, is an appropriate complement to the EOO literature, because it is principally focused on the coordination and adaptation dimensions of purchaser-provider interaction.^20^ It has been suggested that safeguarding is positively reinforced by relational norms and trust, and that the existence of contractual and governance safeguards in turn encourages effective coordination and adaptation.^21^ Moreover, as shown previously in commercial settings, the EOO and IOR literatures are complementary even though each focuses our attention on different units of analysis and different analytical problems.^22,23^ As such, we suggest that these literatures can together assist in the development of a more complete understanding of the policy-making domain in healthcare purchasing.

Accordingly, the intended contributions of this paper are to use a realist literature review both to broaden the discussion of strategic purchasing in healthcare beyond the EOO literature and to provide a framework that offers wider and more comprehensive guidance to public policy-makers. The paper is organised as follows. The next section discusses the review methodology and develops a theoretical interpretation framework based on a number of complementary theories from the EOO and the IOR literatures. Then this theoretical interpretation framework is used to draw insights from a number of studies in the healthcare purchasing literature. In particular, we discuss how these complementary theories might be used to inform 3 objectives identified by the international health policy literature as foundations for the development of strategic healthcare purchasing: patient empowerment; effective government stewardship; and improved provider performance.^6,9^ The paper concludes with a brief discussion of key insights from the review, limitations and avenues for future research.

## Methods

This paper tackles the literature through a theory-based realist review.^24^ This was chosen because the strategic purchasing research domain is associated with varied theories across different disciplinary traditions and is characterized by the influence of context on the utility of these different theories.^22,23^ A realist review approach is theory-based in that its aim is to conceptualise why particular policy or practice mechanisms interact with specific contexts to produce certain outcomes.^25^ Theoretical explanations of this kind are referred to as middle-range in that the specified connections between context, mechanism and outcome are conceptual, but not so abstract as to be unamenable to empirical testing.^26^

These context-mechanism-outcome (CMO) configurations are not deterministic, however.^27^ Room is left for human agency. Mechanisms refer to the underlying theoretical assumptions that actors use to interpret and respond to the ideas and practices associated with a policy, programme or intervention. Realist review is therefore an approach that seems more suited than traditional systematic review to exploring complex social interventions in a range of circumstances, with different underlying mechanisms, and with varied intrinsic beliefs and assumptions.^28^

Realist review is, though, a relatively new method, still being developed and with a fairly small number of exemplar studies.^28-31^ There has been an effort to counter these perceived limitations through the development of quality and publication standards under the Realist and Meta-narrative Evidence Syntheses: Evolving Standards (RAMESES) project.^26^ These standards provide a list of methodological and reporting items expected in a realist review. They also give guidance on the depth and detail of explanation necessary to demonstrate acceptable levels of rigour and robustness.

The first step in a realist review is to develop a theoretical interpretation framework. This framework is then used as a basis to guide application of each of the methodological steps specified by the RAMESES standards: literature scoping and search, text sorting and selection, appraisal, data extraction, analysis and synthesis. The framework developed in this paper brings together 4 middle-range theories from the EOO and IOR perspectives.

### Theoretical Interpretation Framework

Strategic purchasing research is underpinned by a very diverse disciplinary base and by the use of many different theories. Theories commonly associated with this domain are AT, TCE, relational contract theory, social exchange theory (SET), resource dependency theory (RDT), network theory, systems theory, game theory, dynamic capabilities and the resource based view.^32^ This theoretical diversity is unsurprising, and it is assumed that there cannot be a single, unified theory to guide action, given the complex and multi-layered character of purchasing and supply chain phenomena.^33^

As a consequence, research in the strategic purchasing domain has often been focused upon choosing and applying complementary theories in order to answer specific research questions or to understand particular management activities such as outsourcing.^22,23^ The same approach is followed in this paper by focusing on 4 theories in the interpretation framework (see Table 1). These are: AT and TCE from the EOO perspective; and SET and RDT from the IOR perspective. These theoretical choices are guided by an interest in understanding the 3 key relationships shown in Figure 1. In particular, the paper asks what these 4 theories can tell us, first about how these relationships might be structured and safeguarded, and second about how these interactions might be coordinated and adapted, to ensure responsive, equitable and efficient healthcare system performance. These theories were chosen therefore for the high frequency of their use in the purchasing literature,^32^ their relevance to the various policy dimensions of strategic healthcare purchasing,^34^ and for their complementarity to ensure a relatively comprehensive theorisation of safeguarding, coordination and adaptation phenomena.^22^ In line with a realist review approach the discussion that follows draws out the contextual assumptions, key explanatory mechanisms and intended outcomes associated with the 4 chosen theories, before considering the basis of their complementarity.

### The Economics of Organization Perspective

Thisperspective has been heavily used to support notions of strategic purchasing in healthcare.^16^ It is grounded in the seminal works on AT^35,36^ and TCE,^37,38^ and is focused on how organizations should choose cost efficient governance structures to safeguard themselves against the risk of bad behaviour by others. Governance structures refers to the range of ownership, contractual and extra-contractual rules, regulations and protocols that might be used to incentivise and control the behaviour of 1 organization (an agent) acting on behalf of another (a principal). AT and TCE each takes discrete transactions as their analytical focus and they share some basic contextual assumptions. First, both principal and agent are assumed to aim to maximise their individual utility.^39^ Second, it is assumed that an agent might exhibit various forms of opportunistic behaviour, which can impact on the principal’s capacity to achieve their desired objectives.

AT assumes that opportunism can potentially occur in the form of adverse selection (misrepresentation of ability) or moral hazard (lack of effort).^40,41^ These involve an agent exploiting an information asymmetry advantage over a principal to win and undertake a contract on an unfair or misleading basis, and thereby achieve additional profit.^42,43^ TCE focuses on the potential for hold-up, which is a post-contractual situation where an agent is able to stop delivery of a good or service until the principal agrees to a deal more favourable to the agent.^38^ The principal is forced to agree to the agent’s terms, because they are locked-in to the contract by significant and asymmetric sunk cost investments in assets like buildings, machinery or management knowledge.^44,45^

The theories make different assumptions about actor rationality, however. AT assumes that actors are rational and therefore have the capacity to make decisions based on all of the information available to them.^40^ It does acknowledge, though, that information relevant to an interaction between a principal and an agent is not necessarily perfectly or costlessly available to both organizations. One organization might therefore be faced with a situation of information asymmetry.^46^ TCE, by contrast, assumes that actors have inherent bounded rationality. Actors make rational decisions, but within limits imposed by a restricted cognitive ability.^47^

These different contextual assumptions mean the theories propose slightly different mechanisms to explain how the intended outcome of efficiently mitigating the potential for agent opportunism might be achieved. AT is concerned with designing complete contracts to address opportunism by incentivising the agent to make its capabilities explicit and to take appropriate observable action.^40^ By contrast, TCE assumes bounded rationality and therefore that contracts designed ex ante tend to be incomplete.^48^ Thus, to prevent an agent from opportunistically exploiting the gaps in a contract the principal needs to choose efficient forms of extra-contractual governance to incentivise appropriate behaviour. A simple, low cost spot market mechanism is suggested for transactions with a low potential for opportunism, while more complex and higher cost network or hierarchical governance mechanisms are suggested for more hazardous transactions.^38^

Neither of these theories provides an analysis of the dynamics of change between different forms governance, however. Instead they make static comparisons of the efficiency of different governance mechanisms in addressing different transactional circumstances. To understand changes in governance and the associated processes of coordination and adaptation we need to turn to the IOR perspective.

### The Inter-organizational Relationships Perspective

The analytical focus in this perspective is on relationship interactions between organizations and their interaction with a wider network environment. Although these ideas are less commonly deployed in support of notions of strategic purchasing in healthcare than those discussed above, they have been central to work on collaborative IOR across a range of industries and sectors including healthcare.^49-51^ A number of different theoretical strands can be identified within this perspective, but these do share a number of important contextual assumptions: first, that every organization is reliant to some extent on other organizations for the resources that it needs to succeed; second that actors are typically trustworthy, exhibiting simple self-interest rather than opportunism; and third that actors have bounded rationality.

One key strand, the industrial networks view, suggests that organizations are linked through resource dependency and that these linkages are characterised by the exchange of existing resources and the co-creation of new resources through adaptation.^52,53^ The intended outcome explained by these mechanisms of exchange and adaptation is value appropriation and, when possible, value creation through innovation.^54^ This view also draws on SET to explain how IOR operate and evolve over time, using concepts such as cooperation, trust and conflict behaviour.^55,56^ This suggests that a purely economic analysis of relationships, such as that offered by the EOO perspective, is of limited utility because there are also important relational norms such as flexibility and reciprocity that derive from the social context of an exchange.^57^ Relational contract theory offers a close analogue of this argument.^58^ Change in IOR is seen primarily as emergent and it is assumed that relationships are in a constant state of flux. Actors are assumed to have bounded rationality and, consequently, there are limitations on their understanding of the network environment. This in turn places some limits on their capacity to undertake intentionally planned management action to design and reshape the network context.^51^ Planned change is possible, but its consequences cannot be fully foreseen.

Another strand in this perspective uses RDT to examine the concept of power in supply chains.^59^ Like the industrial networks view the power approach proposes that organizations are embedded in a wider network with which they need to interact to achieve their objectives. The intended outcome, again, is to create and capture value through exchange and adaptation processes. This view differs, however, in its strong emphasis on power as the key mechanism explaining how this relationship and network management is expressed.^60^ While the industrial networks view focuses solely on the notion of organizations acting collectively, the power approach argues that the management of a network might sometimes take this mutual form, but on other occasions take the form of a dominant organization directing the behaviour of others.^61^ The power approach therefore proposes that the nature of the power structures underpinning IOR shape the incentives for collaborative interactions to improve network performance.^62^

The theoretical interpretation framework in Table 1 summarises and compares the chosen theories in terms of contextual assumptions, key explanatory mechanisms and intended outcomes. A focus on underlying CMO characteristics shows how these theories can be used in a complementary way. It has been argued that if theories are to be used in combination they must meet 2 key requirements: a focus on the same or relatively close empirical domains; and reasonably congruent or compatible underlying assumptions.^63^

**Table 1:** Theoretical Interpretation Framework

**CMO Characteristics**	**EOO Perspective (AT and TCE)**	**IOR Perspective (SET and RDT)**
Contextual assumptions	Focus of analysis: buyer-supplier transaction Buyers and suppliers have differing motivations and preferences – potential for opportunism Buyers have either bounded rationality or face information asymmetry	Focus of analysis: buyer-supplier relationship and its position in wider network Organizations do not own all of the resources needed to succeed Actors are self-interested and have bounded rationality
Key explanatory mechanisms	Contractual or governance safeguards as mechanism for incentivising, monitoring and disciplining supplier behaviour	Dynamic coordination and adaptation between buyers and suppliers over time Where possible appropriate relationship and network design and management to mitigate dependency on others Emergence in some cases of collaborative, high trust relations
Intended outcome(s)	Mitigation of supplier opportunism at most efficient level of agency or transaction costs	Maximizing value appropriation and, when possible, value creation through collaboration

Abbreviations: CMO, context-mechanism-outcome; EOO, economics of organization; AT, agency theory; TCE, transaction cost economics; IOR, inter-organizational relationships; SET, social exchange theory; RDT, resource dependency theory.

Each of the perspectives is interested in broadly the same empirical domain, inter-organizational interactions, although there are differences in emphasis. The EOO perspective focuses on how these interactions are structured and safeguarded through different governance mechanisms, while the IOR perspective addresses the management behaviours that characterise coordination and adaptation processes. The requirement of congruent or compatible assumptions is also broadly met. Each perspective assumes that actors either have bounded rationality or are not fully informed when making decisions. Each perspective also assumes that organizations will need to interact with others to achieve their objectives. There is a difference, however, between the perspectives regarding their default behavioural assumptions. EOO assumes self-interest seeking with guile (opportunism) is a possibility, while the IOR perspective assumes that simple self-interest typically motivates actors. Even here, though, it is possible to discern broad complementarity. EOO assumes that opportunism is a risk, a possible behaviour rather than a ubiquitous one. This leaves space for simple self-interestedness where the benefits of this behaviour outweigh those of opportunism, what has been called calculative trustworthiness.^38^

### Search Strategy and Selection Criteria

The literature search was undertaken in October and November 2016. Given the demographic of the research team, the search was limited to retrieve texts written in English only. Consistent with the RAMESES standard for literature scoping and search,^26^ 5 scholarly databases were used: ProQuest, EBSCO, Scopus, PubMed, and the Web of Science. These databases were chosen for their comprehensiveness and relevance to the review objective. The search was not limited to a particular time period of publication, because the intention was to be as comprehensive as possible. To ensure quality the search focused primarily on finding peer reviewed journal articles, although research reports, monographs and edited collections of academic papers were also included on the basis that they would have received similar peer review.

Table 2 shows the search terms that were used. Some of these were combined with the Boolean operators AND or OR, which narrowed and widened the search respectively. Some were truncated to capture various relevant suffixes of a term for maximum coverage. Speech marks were used if it was necessary to keep multiple words together as a single search term to further ensure relevance. These terms were generated from 2 sources: the theoretical interpretation framework discussed above and the international policy literature on strategic healthcare purchasing available at the time of our search.^5-9^ In the former category search terms associated with the key CMO characteristics in the EOO and IOR literatures were chosen. In the latter category terms associated with the key policy objectives of patient empowerment, government stewardship and provider performance were chosen.

**Table 2:** Search Categories and Terms

**Category**	**Search Terms**
CMO characteristics in the EOO and IOR literatures	transact*; opportun*; contract* OR governance; ‘contractual safeguards’; ‘governance safeguards’; ‘contracting mechanism’; ‘governance mechanism’; network AND coordinat*; network* AND collaborat*; collaborat*; coordinat* AND adapt*; coordinat*; adapt*; collaborat* AND trust; trust; power OR dependen*
Policy objectives in strategic healthcare purchasing	health* AND purchas*; health* AND procur*; health* AND commission*; ‘patient empowerment’; ‘needs assessment’; consult*; choice; ‘health strategy’; ‘health targets’; ‘healthcare regulation’; provider AND performance; ‘provider autonomy’; purchas* AND accountab*; provider AND accountab*

Abbreviations: CMO, context-mechanism-outcome; EOO, economics of organization; IOR, inter-organizational relationships.

**Figure 2:**
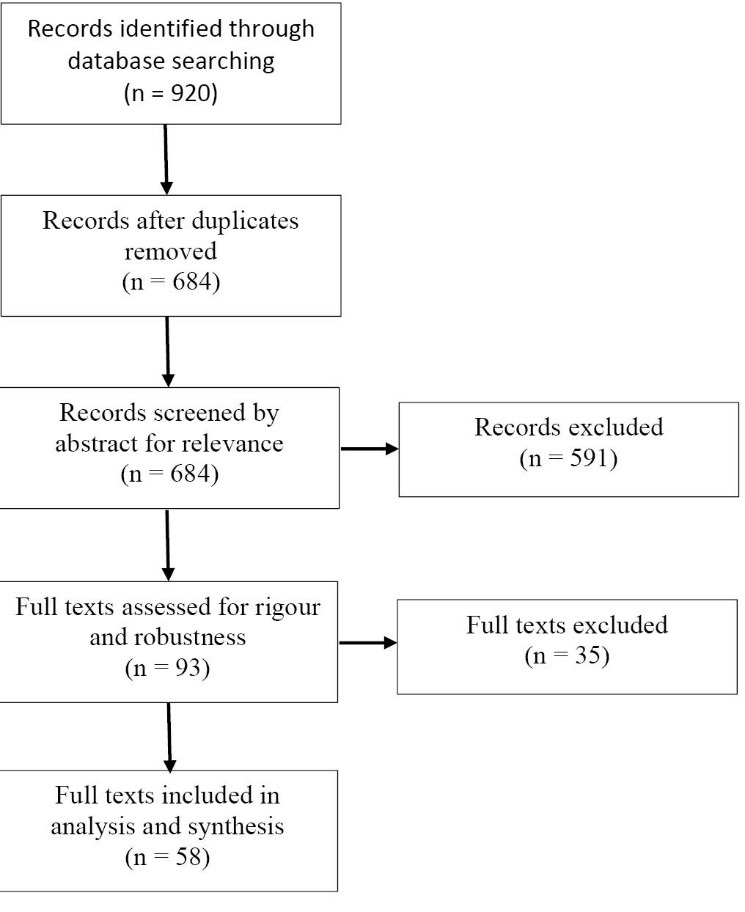
Flow Diagram of Search Results.

References for all 920 texts found by the initial search were entered into an EndNote Library. Consistent with the RAMESES standard, the text sorting and selection strategy was based on purposive sampling to focus on literature that addressed the CMO characteristics and policy objectives of interest.^63^ A 2-stepapproach was used: first an initial sift to remove duplicates (236 records removed) and then screening of abstracts to ensure broad relevance. Two exclusion criteria were applied in the screening of abstracts. Papers were excluded either if they dealt with consumer rather than organisational purchasing topics, or if they were purely theoretical or non-empirical; this process removed a further 591 records. A third exclusion criterion was then applied in the next methodological step, appraisal of full texts. Papers were excluded if they lacked a sufficiently rigorous and robust evidence base, such as quantitative research with an inadequate sample size or qualitative research reporting anecdotal evidence. Thirty-five records were removed at this step, which resulted in a total of 58 full texts being identified for final analysis and synthesis. A flow diagram of the search results is given in Figure 2.

### Analysis

The analytical process involved first extracting key findings or insights from each text and categorising these in terms of the 3 policy objectives associated with strategic healthcare purchasing, ie, patient empowerment, government stewardship and provider performance. The findings in each of these categories were then analysed and interpreted through the lenses of contextual assumptions, explanatory mechanisms and intended outcomes associated with the EOO and IOR theories. This was done using a coding schema developed from the theoretical interpretation framework. Finally, this coded textual data was synthesized to draw out broader lessons for strategic purchasing in healthcare.

## Results

This section explores what lessons relevant to patient empowerment, effective government stewardship, and improved provider performance can be inferred from applying the theoretical interpretation framework to the literature reviewed. Table 3 provides a summary of these lessons. The 58 texts analysed report evidence from 28 countries, of which 26 are high income, 1 upper-middle income (Iran) and 1 lower-middle income (India). The most studied countries are all high income: England, the United Kingdom, the Netherlands, Germany, the United States, and New Zealand.

**Table 3:** Lessons From Complementary Theories to Guide Strategic Purchasing in Healthcare

**Policy Objectives**	**EOO Perspective (AT and TCE)**	**IOR Perspective (SET and RDT)**
Patient empowerment		
Needs assessment and patient consultation		Purchasers need to build trust with citizens and patients to gain access to information about their needs and wants, eg, purchasing organisations (CCGs) in English NHS designed to involve clinicians in purchasing decisions and to build on trust between patients and primary care physicians. Information needed to facilitate system responsiveness and the co-creation of value through better tailored services. To build trust purchasers must avoid being dominated by powerful citizen, patient or industry groups.
Purchaser accountability	Need to establish clearly defined rights for patients and responsibilities for purchasers. Legal enforcement useful, but public awareness of a code or charter can be enough to discipline purchasers through perceived social obligations.	Rights and responsibilities must be enacted in working custom and practice through regular patient-purchaser interaction.
Patient choice	Need blend of market governance for standardized, low complexity treatments (purchaser as supportive intermediary), and network governance for more bespoke, high complexity, on-going treatments (purchaser as lead decision-maker).	On-going patient-purchaser interaction vital to build trust and cooperation, particularly where service needs require specific adaptations.
Effective government stewardship		
Formulation of national health strategy	Strategy needs to contain clear, specific targets to incentivise purchasers to align their decisions and behaviours with national policy objectives. Targets need to be designed with awareness of potential for opportunistic behaviours by purchasers. Need therefore to be stretching, transparent and evidence based, and linked to clear performance management systems.	To gain access to local knowledge about needs and priorities, and thereby set realistic targets, government needs close, trusting relationships with purchasers. Targets need to blend national and local insights to encourage innovation and avoid resistance at local level. Government advised to build cooperative alliances with purchasers to find compromises between national priorities and potentially competing local interests.
Regulation	Regulation needs to cater for possibility of opportunistic behaviour (clear minimum standards and monitoring mechanisms), but also leave room for development of goodwill trust.	Regulation needs to be broad and integrated to recognise that purchasing occurs in an interconnected network of patients, purchasers and providers – if too narrowly focused, likely to produce unbalanced outcomes, eg, the statutory duty of NHS England to annually assess the success of purchasers in building relationships with other actors in their local health systems (patients, providers, local government, community and voluntary groups).
Improved provider performance		
Provider autonomy	Provider decision autonomy may drive innovation and efficient resource use, but may also create scope for opportunistic behaviour.	
Provider accountability mechanisms	Purchasers advised to use an appropriate blend of contractual and extra-contractual governance mechanisms to mitigate potential provider opportunism. Contracts are best mechanism for purchasers to specify, monitor and enforce requirements, and to set out performance improvement targets, where services are relatively standardized and low complexity, eg, CQUIN incentives used in English NHS contracts to make a proportion of healthcare providers’ income conditional on demonstrating improvements in quality and innovation in specified areas of patient care. For more complex, longer-term and bespoke services, contracts tend to be incomplete and need to be complemented with extra-contractual governance mechanisms.	
Purchaser-provider power structure		Provider’s performance and willingness to improve are significantly influenced by prevailing power structure and dynamics of change in that structure. May be very difficult for a purchaser to work with a dominant provider, irrespective of whether it is public, private or third sector. May also be problematic for a smaller provider to work with a more powerful purchaser. Interdependence likely to be best power structure to incentivise collaboration needed to drive innovation and performance improvement. For purchasers to achieve interdependence with relatively autonomous providers they need to be enabled to develop countervailing power resources.

Abbreviations: CMO, context-mechanism-outcome; EOO, economics of organization; AT, agency theory; TCE, transaction cost economics; IOR, inter-organizational relationships; SET, social exchange theory; RDT, resource dependency theory; CCGs, clinical commissioning groups; CQUIN, commissioning for quality and innovation; NHS, National Health Service.

### Patient Empowerment

This policy objective concerns the patient-purchaser relationship. The main focus here is on health system responsiveness to patient needs and expectations. Needs and expectations are clearly not the same, however, and patients will often expect more than they need. This requires purchasers to make trade-offs to balance the demands of different patient groups. To the extent that purchasers are able to meet the needs of a broad patient population, though, equity of access and allocative efficiency should also be achievable. According to the work of Figueras et al^9^ there are 4 policy avenues through which patients can be empowered: assessment of population health needs at an aggregate level; purchaser consultation with patients to better understand their views regarding purchasing priorities; mechanisms to ensure that purchasers are accountable to patients; and increasing patients’ choice of providers.

Assessment of population health needs and consultation with patients both involve a dynamic interaction process, which is best interpreted through the IOR perspective. This interpretation suggests that data about population health needs or the views of patients can be seen as resources required by purchasers to enable them to be more responsive, and that by making purchasing decisions that better reflect the needs and priorities of patients, value is being co-created.^65,66^ It also emphasizes the importance for purchasers of building trust with citizens and patients to facilitate effective access to data about needs and priorities through on-going consultation.^67^ A key aspect of building trust is to ensure that more powerful advocacy groups are not given preferential lobbying access to purchasers. An awareness of the role of power in IOR suggests that purchasers need to avoid being dominated by certain patient or citizen groups, either those that are better organised or that have been co-opted by industry interests.^68,69^ System responsiveness, equity and allocative efficiency can all be compromised where a powerful advocacy group dominates purchasing decisions.

Accountability mechanisms, which typically involve the setting up of governance structures, some with legal authority, can usefully be interpreted through the EOO perspective. These mechanisms include: giving patients formal representation in purchasing organisations^70^; statutory packages of care with coverage guarantees^71^; patients’ rights legislation^72^; and formal complaints procedures.^73^ The EOO perspective suggests that these governance mechanisms are likely to work well where they establish a clearly defined set of rights for patients and responsibilities for purchasers, akin to a complete contract. Another important consideration is whether purchaser accountability is codified in legislation and is therefore legally enforceable.^74^ This is not always the case. In some countries rights and responsibilities are presented in the form of a patients’ charter or a code without legal force.^72^ Even without a legal dimension, however, such a charter may act as a useful means of creating perceived social obligations that discipline purchaser behaviour by raising public awareness of required standards of care.

Ultimately, though, creating governance structures to codify rights and responsibilities is only part of what is required to ensure meaningful patient empowerment. To ensure that these commitments are more than merely symbolic they need to be enacted in working custom and practice.^75^ This suggests that policy-makers might draw also on the notion of dynamic exchange and adaptation offered by the IOR perspective. Using this lens suggests that patients and purchasers might be encouraged interact with one another on a regular basis to learn and interpret how their rights and responsibilities can be exercised and might play out in practice.^76^

Turning to policies to increase patients’ choice of providers, the EOO perspective suggests that where a market governance structure is possible, with relative ease of switching, this can provide high-powered incentives for providers to be responsive to patient needs and expectations. TCE suggests that this kind of governance is appropriate where a healthcare service is relatively standardised or treatments are fairly simple so patients are able to make well informed choices. Minor surgical procedures or non-specialist home care services would have these characteristics.^77^ Here, the purchaser’s most appropriate role might be to act as a supportive intermediary, providing patients with information about providers to facilitate choice and acting as an ultimate safeguard to ensure that providers meet their contractual commitments where disputes arise.^78^

In the case of many healthcare services, however, treatments are much more complex and specialised, there is a much higher level of service adaptation to the particular needs of individual patients, and patient needs are dynamic across an extended period of time. The treatment of chronic, long-term conditions like diabetes or heart disease or the provision of specialist home care services would have these characteristics.^79^ Here, TCE suggests that market governance is unlikely to be an effective means of achieving patient choice, because of uncertainty associated with complex and dynamic patient needs and specific sunk cost investments needed for providers to adapt their services. Instead a network or collaborative form of governance is seen as more appropriate. This means that patients might be expected to rely more on purchasers to make provider choices and to manage the specific details of service delivery on their behalf, because the potential hazards of provider opportunism and the negative consequences of poor performance are greater.^78,80^ In order to maintain patient empowerment in a network structure, however, it is important for purchasers to undertake meaningful consultation with patients on issues around service design and delivery.^81^

The IOR perspective also emphasizes the importance of enabling patients to remain actively involved in decisions where a purchaser is choosing and managing providers of complex, specialised services on their behalf. In this case, though, a close interaction is interpreted as a means of building trust and cooperation between patients and purchasers rather than as a means safeguarding the patient against provider opportunism.^82^ Value should, in turn, be co-created through the exchange of information to enable appropriate service adaptation.^65^

### Effective Government Stewardship

This policy objective is concerned with the government’s responsibility for steering the decisions and activities of healthcare purchasers. The literature suggests 2 key sets of activities that play a part in stewardship, which apply both in national health systems with public sector purchasers and in social health insurance systems with private sector purchasers. First, government formulates a national health strategy that provides policy direction for purchasers to help them allocate resources to appropriate health interventions in line with the population’s needs and priorities.^83^ This strategy thereby delivers a responsive and equitable health system. Second, government creates an appropriate regulatory framework to ensure purchaser accountability and responsiveness, equity of patient access to healthcare and efficient resource utilisation.^84^

Turning first to national health strategy, the EOO perspective suggests a need for this to go beyond vague aspirations and use specific, well-defined targets linked to national policy objectives. In this interpretation, targets are a form of complete contracting as envisaged by AT that incentivises purchasers to align their individual decisions and behaviours with the government’s broader objectives for the healthcare system. Evidence shows however that where such targets have been adopted experience of implementation has been mixed.^85^

Various insights have been drawn from these mixed experiences. First, it is suggested that while stretching targets are more likely to encourage improvement and innovation, care also needs to be taken to ensure that targets are technically realistic and culturally legitimate to enable implementation and avoid demotivation of purchasers.^85^ Second, targets need to be transparent and evidence based to assist measurement. Third, it is suggested that targets need to be integrated into performance management systems and contracts alongside appropriate payment incentives to ensure implementation.^86^ This needs to be done, however, with an awareness of possible negative behaviours such as tunnel vision, myopia and gaming.^87,88^ As AT suggests, a principal may often suffer from an information asymmetry relative to the agents acting on its behalf and this creates the potential for various forms of opportunistic behaviour. In this interpretation, the government as principal might not have complete information about the resource allocation decisions and behaviours of healthcare purchasers. There is a potential, therefore, for purchasers to make decisions that are highly responsive to local health priorities, but only pay limited attention to national priorities and guidance and thereby undermine equity of patient access across the health system as a whole.^89^

These insights suggest a need to appreciate the broader social and behavioural context within which targets are being set. This highlights the relevance of theories from the IOR perspective, drawing on concepts such as power, resource dependency, and trust. The need to ensure that targets are technically realistic and achievable emphasizes that it is important for the government to work with the healthcare purchasers responsible for meeting those targets. The IOR perspective suggests that healthcare purchasers can provide valuable resources to national government in the form of local knowledge about population needs and priorities, and that trusting relationships are required to facilitate government access to that knowledge.^89^ In practice, this means that government imposition of top-down national targets is likely to disenfranchise healthcare purchasers, stifle innovation and potentially provoke resistance at a local level.^90^ Evidence suggests that national strategy formulation blended with local input is likely to prove more effective.^91^

The IOR perspective also draws our attention to the potential for an unequal balance of power between national governments and healthcare purchasers. National governments are typically reliant on various types of regional and local purchasers, either public or private, to implement their health strategy.^92^ This dependency means that even the most clearly defined strategy can be undermined or subverted by purchasers that do not support it.^90^ This suggests that a key element of stewardship is likely to involve national government building alliances with purchasing organisations to find ways of achieving a consensus that better aligns national policy objectives with potentially competing local interests.^93^ A strategic programme approach to healthcare purchasing has been suggested as 1 mechanism to achieve such a consensus.^86^

The second key aspect of the government stewardship role is the creation of a regulatory framework that stands in place of hierarchical management control where there is a purchaser-provider split. Two broad insights for regulation are offered by our complementary theories.

First, it is suggested that regulation needs to achieve an appropriate balance between compliance and deterrence.^94^ Compliance regulation has a positive intent in that it is designed to encourage good behaviour by purchasers. The underlying behavioural assumption here is that suggested by the IOR perspective. Purchasers are seen as fundamentally trustworthy and will do the right thing if they are appropriately supported. The role of the regulator in this perspective is that of a critical friend, providing guidance and advice while leaving room for innovative and entrepreneurial behaviour in the selection and management of healthcare providers. Deterrence regulation, by contrast, is grounded in the behavioural assumption of the EOO perspective. Where purchasers are allowed to retain any financial surpluses from their purchasing activities they may have an incentive for opportunistic behaviour, for example through choosing lower price and poorer quality providers, and so detailed and demanding standards are required, backed up by a rigorous monitoring and enforcement regime. The danger, of course, is that such deterrence regulation becomes too onerous and punitive and stifles innovation by purchasers who are inherently trustworthy.^95^ As we noted earlier, the EOO perspective argues that opportunism is a possible rather than a ubiquitous behaviour. The lesson suggested here then is that the government’s regulatory framework ought to cater for the possibility of opportunism by setting out clear minimum standards and monitoring mechanisms, while also leaving some freedom from detailed oversight to encourage purchasers to innovate. This suggests a broad balance in favour of compliance regulation.

Second, the regulatory framework needs to be sufficiently broad and integrated to address the multiple desired outcomes of health system responsiveness, equity of patient access and efficient resource use, in a coordinated way. Drawing on the IOR perspective, we can suggest that regulation needs to be designed in a way that recognises that healthcare purchasing occurs in an interconnected network of patients, purchasers and providers,^96^ and that the activities, espoused values and underlying assumptions of these different actors are continuously impacting in complex recursive ways on responsiveness, equity and efficiency.^97^ As such, a regulatory framework that is narrowly focused on discrete artefacts of the purchasing system (eg, contracts or payment mechanisms) or on purely economic concerns (eg, cost control) is likely to deliver unbalanced outcomes. Consequently, it makes sense for regulation to consider a range of domains.

Four possible domains are suggested by the literature. First, regulation might ensure that purchasers are accountable to patients through various mechanisms to provide information, facilitate participation in purchasing decisions, and set out rights and means of redress.^70,72^ Second, regulation might ensure that purchasers are accountable to government for the efficient and equitable use of insurance premiums or taxpayers’ money in the purchasing of healthcare services.^98^ Third, regulation might act to ensure fairness and transparency in the commissioning and contracting processes that take place between purchasers and providers.^99^ Finally, regulation might focus on ensuring that providers are safe and competent to deliver healthcare of the required quality.^100^

### Improved Provider Performance

The efforts of purchasers to foster improved provider performance are related to the delivery of greater value, broadly defined, to patients and citizens. For some authors this greater value is most obviously associated with the drive to achieve efficient utilisation of health system resources – the pursuit of better value for money in the delivery of healthcare in the form of increased quality while controlling the growth of costs.^19^ According to others, however, value is expressed in terms of health system responsiveness and equity of patient access, which are also intrinsically determined by the decisions and behaviours of healthcare providers in response to the activities of purchasers.^101,102^

A key challenge for purchasers is that system responsiveness, equity of access and resource efficiency might be in conflict with one another. For example, consolidation of a service in 1 main location to enhance cost effectiveness and increase quality is very likely to have an adverse impact on equity of access for patients living further away. Decisions aimed at improving provider performance will therefore require purchasers to make trade-offs. The literature identifies 3 factors that might influence the response of providers to purchasing decisions and, in turn, determine how these trade-offs are expressed in practice.

The first is the degree and types of autonomy that providers have when they are deciding how to meet the requirements of purchasers. Policy-makers might give providers autonomy over a number of significant decision areas, for example: staffing (numbers and skill mix); financial management (ability to take loans); the scope of activities (which services are offered and where); and capital investment (size and location of buildings, technology mix).^103^ The rationale for granting such autonomy is based on the idea of creating market competition between different types of providers (public, private and third sector), and incentivising innovative and efficient choices by providers by giving them a right to retain ‘surplus’ resources.^104,105^ The primary emphasis here is on efficient resource utilisation, although policies of this type are also intended to enhance quality of care and responsiveness to patient needs.^102^

The EOO perspective draws attention however to the potential of such provider autonomy to create scope for opportunistic behaviour that might damage system responsiveness, equity of patient access and efficiency of resource use.^106,107^ For instance, if providers are able to choose how and where a service is delivered they might choose to indulge in moral hazard to enhance their surplus. This might take the form of quality shading (using fewer or less well qualified staff), shirking (only partially carrying out certain tasks) or cream skimming (choosing to focus on less risky and less costly treatments and categories of patients).

This insight suggests that a second key factor likely to shape provider behaviour and performance are the kinds of governance mechanisms used to make providers accountable to purchasers, and the effectiveness of those mechanisms in mitigating opportunism. Again drawing on the EOO perspective, a broad lesson is that purchasers need to create an appropriate blend of contractual mechanisms and extra-contractual governance to manage providers in a transaction cost efficient way.^107,108^

AT suggests that purchasers use various contractual and payment mechanisms to specify, monitor, and enforce their requirements on delivered volumes of care and quality standards.^5^ Contracts are also suggested by AT as the best vehicle for setting performance improvement targets and monitoring the extent to which these have been achieved.^78,109^ The use of contracts to manage the behaviour of a provider relies on an assumption that the purchaser is capable of accessing all of the information needed to design a complete agreement, which will mitigate potential provider opportunism. In other words, the purchaser is assumed to be rational, although there are costs associated with being fully informed. TCE offers a more nuanced interpretation of decision-maker rationality, suggesting that bounded rationality limits the purchaser’s capability to draw up a complete contract for all but the simplest, one-off patient needs. TCE argues therefore that where a patient’s needs are more complex and longer-term, contracts tend to be incomplete and need to be complemented with various extra-contractual governance mechanisms.^106-108^ The objective of such mechanisms, for example joint relationship-specific investments or the posting of a financial bond against contract violation, is to create a balanced relationship between purchaser and provider and thereby to dis-incentivise provider opportunism.

The third and final factor likely to impact on how providers respond to purchasing decisions picks up on this idea of creating a balanced relationship. More specifically, we are interested in the balance of power between purchasers and providers and the moves that largely autonomous providers, whether they are public, private or third sector, might make to protect or enhance their power relative to purchasers.^104^ Such moves can either be structural (eg, mergers or collaboration between providers) or tactical (eg, offering services to a wider range of purchasers).^103^

RDT from the IOR perspective suggests that a provider’s performance and its willingness to improve are significantly influenced by the prevailing balance of power and the dynamics of change in that balance over time. So, where a purchaser is seeking to improve the performance of a provider through, for example, information sharing and service redesign, RDT suggests that moves by the provider to create or maintain a position of dominance might create barriers to the desired improvement. A dominant provider is likely to resist or subvert changes requested by a purchaser where those changes are perceived as damaging to its interests.^110^ Similarly, if a purchaser is seen as too powerful by smaller third sector providers they are unlikely to want to share ideas for service improvement for fear that the purchaser will simply pirate those ideas and use them as part of a competitive tendering process involving other providers.^101^ Rather, it is argued that purchaser-provider collaboration and the development of trust to support performance improvement is best incentivised by interdependence, a balanced and committed power structure.^111,112^ The broad lesson for policy-makers is that purchasers dealing with relatively autonomous providers need to be enabled to develop countervailing power resources if they are to achieve interdependence.^49^ As they are for providers, these countervailing resources might be a consequence of structural moves (eg, mergers or collaboration between purchasers),^113^ or tactical moves (eg, targeting additional financial rewards on certain providers to encourage desired service improvements).^114^

## Discussion and Conclusion

Other authors have argued that using policy learning to translate policies from one context to another requires a realist explanation of what works, for whom, under what circumstances and why.^27^ This, in turn, requires a conceptualisation of CMO configurations, with theory being used to explain what outcomes are likely to follow if a particular policy is introduced in a certain context. Realist review is about reflecting on the explanatory scope of different theories to develop contextually sensitive guidance rather than to make universal rules.^25^ In this paper realist review is used to explore 4 theories as bases for drawing lessons from the empirical literature to guide the pursuit of policies intended to make healthcare purchasing more strategic. Specifically, the chosen theories are used to think about what kinds of safeguarding mechanisms and coordination and adaptation behaviours might be needed to pursue patient empowerment, effective government stewardship and improved provider performance, 3 key policy objectives suggested by the literature as foundations for strategic healthcare purchasing.^6,9^

The findings of this review reinforce 2 significant observations made elsewhere in the literature. First, there is no single, unified theory that can explain strategic purchasing, nor is there ever likely to be one given the broad and multifaceted nature of this research domain.^33^ This suggests that different theories should be used in a complementary way to provide a more complete understanding.^22^ Second, the analysis suggests that policy-makers face choices about which theory might work best as a basis for interpreting their situation and for guiding their policy decisions.^23^ So, for instance, the EOO perspective is most useful and relevant for learning about the safeguarding dimensions of strategic healthcare purchasing. It provides insights for understanding how interactions between patients, purchasers, government and providers might be structured, controlled and incentivised to deliver responsive, equitable and efficient healthcare. The IOR perspective, by contrast, is most useful for learning about the relationship coordination and adaptation dimensions of strategic purchasing. It assists with understanding how the resources offered by each of the key groups in healthcare might be managed through dynamic processes of exchange and adaptation to achieve valued outcomes. It also emphasizes that the behaviours of actors within any health system have an embedded, social character and are not motivated simply by economic calculation. Trust and collaboration matter too.

We agree with the argument that theory-based policy learning is enormously challenging given the complexity of the social systems within which policy interventions are made.^14^ We do not believe, however, that the best way to address this challenge of learning within complexity is to use a narrow conceptual frame of reference. We have argued that the EOO perspective, which is heavily used in analyses of healthcare purchasing, does take us some of the way in understanding how to achieve strategic purchasing in healthcare, but as we have suggested IOR theories are needed too. As Van Raaij^10^ observes, key ideas and concepts from the IOR perspective, such as trust and collaboration, have historically received much less attention in the international health policy literature than EOO concepts like opportunism and contractual safeguards. We contend that our theoretical interpretation framework represents a vital step towards redressing the balance and offering a broader understanding.

This framework has limitations, however, because we have focused our attention on deriving general, theoretically-informed lessons for strategic purchasing. For instance, the framework lacks a temporal dimension so it is unable to show if the relative utility of each theoretical perspective as a basis for action might change over time as the characteristics and demands of a healthcare purchasing situation change. To tackle this limitation, further work could be done to apply the framework to a range of newly established and mature purchaser-provider relationships. We have also not yet considered how these lessons might play out in specific institutional or geographical contexts; moreover, the studies covered in the review are heavily focused on experiences in a handful of high-income countries, particularly England and the United Kingdom. A greater diversity of empirical work needs to be reviewed then to explore how far these lessons are a practically useful guide to develop strategic purchasing in a wider variety of healthcare systems and country settings. For instance, recently published findings from the RESYST Project^115^ could be interpreted on the basis of our framework to identify how these lessons apply to healthcare purchasing in low- and middle-income countries such as Thailand,^116^ Nigeria,^117^ and Tanzania.^118^ We could also identify a number of potential contextual variables to be transposed onto the actors and the relationships described in Table 3. The literature suggests, for example, that purchasers could be differentiated by the scope (local or regional) or the focus (population groups or health conditions) of their responsibilities, and that these differences might have a significant impact on how patients are consulted about their needs,^64-67^ on how national governments undertake their stewardship role,^89-91^ and on how providers are encouragedto improve their performance.^48,109,112^

## Acknowledgements

This work was supported by the Health Services & Delivery Research programme of the UK National Institute for Health Research [grant No. 12/5004/03]. The NIHR had no role in the conduct of this research. The views and opinions expressed are those of the authors and do not necessarily reflect those of the NHS, the NIHR, NETSCC, the HS&DR programme or the Department of Health.

## Ethical issues

Not applicable.

## Competing interests

Authors declare that they have no competing interests.

## Authors’ contributions

All authors conceived the idea for the review through discussion. JS undertook the literature search, extracted and analysed the data and wrote the first draft of the manuscript. All authors provided input into ongoing iterations of the manuscript and approved the final version.

## Authors’ affiliations

^1^Birmingham Business School, University of Birmingham, Birmingham, UK. ^2^Health Services Management Centre, University of Birmingham, Birmingham, UK.
